# Microbe on the move: *Akkermansia* in infectious diseases and emerging roles in gynecological health

**DOI:** 10.1128/jb.00491-25

**Published:** 2026-04-30

**Authors:** Stephanie M. Marroquin, Kelly S. Doran

**Affiliations:** 1 Department of Immunology and Microbiology, University of Colorado School of Medicine 12225, Aurora, Colorado, USA; National Institutes of Health, Bethesda, Maryland, USA

**Keywords:** female genital tract, gastrointestinal tract, pregnancy, gynecological health, infectious disease, microbiota, akkermansia

## Abstract

The genus *Akkermansia* was first described in 2004 following the identification of *Akkermansia muciniphila*, a Gram-negative, mucin-degrading bacterium of the intestine that constitutes 1–3% of the total adult fecal content. Since the interest in *A. muciniphila* in human health has increased over the past decade, an extensive amount of research examining the impact of *A. muciniphila* on metabolic disorders, non-communicable diseases, and during infection has been published. Furthermore, a rapidly evolving area of research is the role of *A. muciniphila* in gynecological health. Many studies have shown that the presence of *A. muciniphila* may decrease the chances of negative health outcomes. Some of these protective effects include enhancement of epithelial barrier integrity and metabolism, immune modulation, and attenuation of inflammatory responses. As such, *A. muciniphila* has gained significant interest for its promising role as a next-generation probiotic. Notably, most of the *in vivo* evidence reviewed here demonstrates the probiotic potential of *A. muciniphila*. However, some findings suggest that its role is context-dependent, which may be influenced by the type of infection, diet, and microbiota composition. Herein, we review associations between *Akkermansia* species and an array of infectious diseases caused by diverse pathogen classes, including bacteria, viruses, fungi, and parasites. We also review the impact of *Akkermansia* species in gynecological conditions, particularly during pregnancy. The emerging role of *A. muciniphila* in promoting health, and in some cases disease, has important implications for understanding complex microbial-host interactions, as well as for the development of novel therapeutics.

## INTRODUCTION

**Fig 1:**
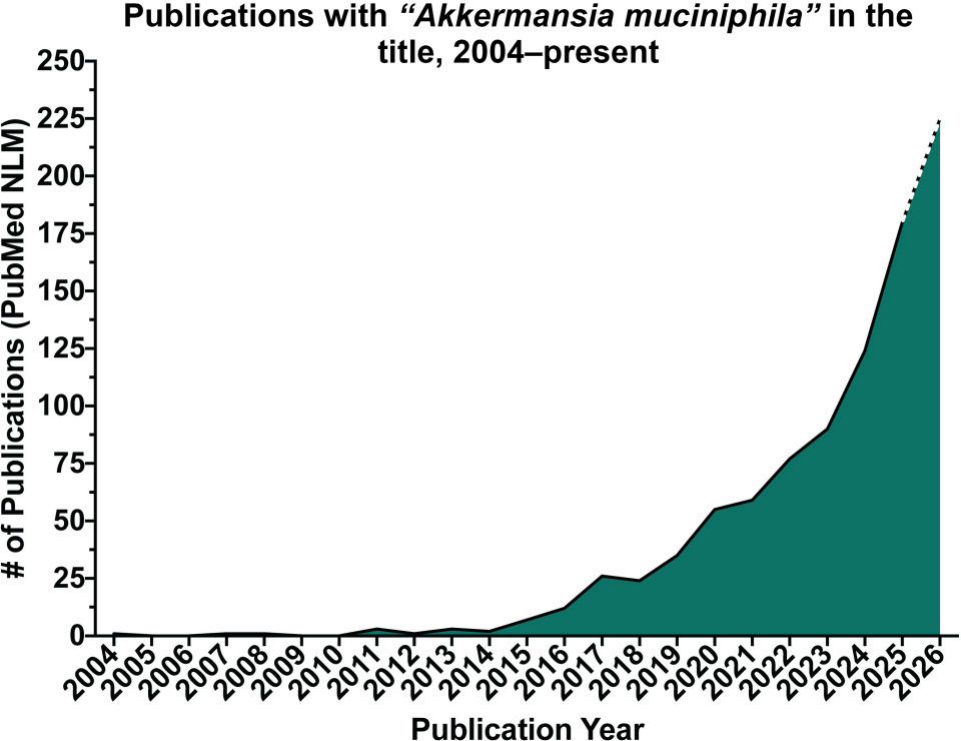
Publications with *Akkermansia muciniphila* in the title from 2004 to present. This graph depicts literature counts for publications containing “*Akkermansia muciniphila*” in the title, which were obtained through a search on PubMed (National Library of Medicine) using Endnote 21. These counts exclude corrections and retractions and demonstrate a predicted final count for 2026 based on a quadratic trend model.

In 2004, *Akkermansia muciniphila* (Muc^T^; ATCC BAA-835) was isolated from human feces using mucin as the sole source of carbon and energy by researchers at the Laboratory of Microbiology, Wageningen University, in the Netherlands (1). *A. muciniphila* is a Gram-negative, non-motile, and non-spore-forming anaerobe that uses mucin as its preferred source of carbon and nitrogen (2). Notably, the discovery of *A. muciniphila* became the first account of members of the phylum Verrucomicrobiota to inhabit the human gut (3). In 2007, the presence of *A. muciniphila* was examined across various stages of the lifespan (4). It was detected in the gastrointestinal (GI) tract as early as 1 month old in infants and steadily colonized within the first year of life, accounting for 1–3% of the gut microbiota in adults (5). Subsequently, about six years after its discovery, the first genome of *A. muciniphila* was sequenced and began to be studied in the context of human health (2, 6, 7). Following a search on PubMed (National Library of Medicine) for publications specifically containing “*Akkermansia muciniphila*” in the title, only 12 articles were shown to be published in its first decade following its identification. Yet, in the last 10 years, there have been around 700 publications focusing on the study of *A. muciniphila* in various contexts, with 304 published in 2024–2025 (Fig. 1). The majority of findings identified that changes in abundance of *Akkermansia* spp. were associated with certain disease states and shortly evolved into examining the use of *A. muciniphila* as treatment to improve health and disease outcomes (7–12).

To date, an extensive amount of publications (approximately 1/3 of the articles featured in Fig. 1) has explored the association of *Akkermansia* spp. or the therapeutic role of *A. muciniphila* in metabolic health, including examination of diseases such as obesity and type 2 diabetes, where *A. muciniphila* has been linked to improved outcomes (9, 13, 14). Notably, prior work bridged mechanistic findings from murine models with outcomes from the first controlled human intervention study (15, 16). In both studies, administration of live and pasteurized *A. muciniphila* was associated with improvements in intestinal barrier function, reductions in circulating lipopolysaccharide (LPS) levels, and other metabolic benefits. Pasteurized *A. muciniphila* demonstrated effects comparable to live bacteria in both mice and humans and in some cases enhanced the beneficial metabolic outcomes in the murine model (16). Plovier et al. further identified Amuc_1100, a thermally stable outer membrane protein that engages TLR2 signaling and remains active following pasteurization, demonstrating a protein-driven mode of action independent of bacterial viability (16).

Given that the GI tract is its natural niche, *A. muciniphila* has also been studied for its role in GI health, including irritable bowel disease, ulcerative colitis, and Crohn’s disease (17–19). Furthermore, microbiome analyses have shown that the relative abundance of *Akkermansia* spp. (depletion in most cases) has not only been associated with improved outcomes of GI tract cancers, such as colorectal cancer, but also in lung, skin, and prostate cancers (20–27). The impact of *Akkermansia* spp. on cardiovascular health has also been examined, including associations with cardiovascular function, abdominal aortic aneurysms, and atherosclerosis (28–30). A large proportion of these studies have shown that *Akkermansia* spp. profoundly improve epithelial gut barrier integrity and mitigate inflammation, and their immunomodulatory role has been linked to anti-inflammatory effects (6, 31–34). Given the amount of work that has been conducted on these aforementioned topics, there is an extensive amount of published reviews that nicely address the impact of *Akkermansia* spp. and *A. muciniphila* on these metabolic disorders and non-communicable diseases (18, 35–44). More recently, research has expanded to examine the role of *Akkermansia* spp. in various infectious diseases, as well as some diseases pertaining to gynecological health (Table 1).

**TABLE 1:** List of infectious and gynecological diseases/ health outcomes that have been associated with *Akkermansia* spp. presence or *A. muciniphila* treatment as a major finding^*a*^

Disease/health outcome	First reported as major finding	Sequencing, experimental model, or both	Study site/treatment method	Findings/role of *Akkermansia* spp. or *A. muciniphila*
Infectious diseases				
*S*. Typhimurium infection	2013	Murine (C3H) Gnotobiotic SIHUMI model	Gut/gastric gavage (live; ATCC BAA-835)	Increased histopathology and inflammation of cecum and colon Increased pathogen load in mesenteric lymph nodes
	2023	Murine (C57BL/6J) Streptomycin- treated model	Gut (live and pasteurized; ATCC BAA-835)	Observed with pretreatment Reduced susceptibility to infection Enhanced gut barrier
*C. difficile* infection (CDI)	2016	Human fecal samples	Gut/n.a.	Increased in CDI patients
	2022	Murine (C57BL/6) CDI model	Gut/oral gavage (live; ATCC BAA-835)	Reduced colon damage and colon epithelial injury Improved local and systemic anti-inflammation Reduced CDI-induced inflammation
	2023	Human cell line infection	Gut (live, UV-killed, supernatant, and extracellular vesicles; ATCC BAA-835)	Reduced colon damage and colon epithelial injury Improved local and systemic anti-inflammation
Schistosomiasis (*S. mansoni*)	2018	Murine (Swiss Webster) fecal samples	Gut/n.a.^*c*^	Expansion during *S. mansoni* infection
*P. gingivalis* infection	2019	Bone marrow macrophages (BMMs), human gingival epithelial cells (hGECs), and murine model of calvarial abscess and periodontitis	Oral and gut/oral administration and gastric gavage (live/pasteurized, purified protein; ATCC BAA-835)	Decreased inflammatory cell infiltration and bone destruction Reduced alveolar bone loss Anti-inflammatory effect on BMMs Increased junctional integrity markers in hGECs
Malaria infection (*P. chabaudi chabaudi* AS)	2019	Murine fecal samples	Gut/n.a.	Negatively associated with parasitic burden
Cerebral Malaria (*P. berghei*)	2019	Murine fecal samples	Gut/n.a.	Increased abundance during *P. berghei* infection
Mucormycosis (*M. circinelloides* infection)	2019	Murine (BALB/C) fecal samples	Gut/n.a.	Decreased abundance in infected mice
Lymphocytic choriomeningitis virus (LCMV)	2020	Murine (C57BL/6) fecal samples	Gut/oral administration (live; ATCC BAA-835)	Increased abundance in mice with fast-spreading and persistent LCMV (via CD8 T cells/CD8 T cell anorexia) Treatment reduced CD8 T cell responses
COVID-19 (SARS-CoV-2 infection)	2021	Human fecal samples	Gut/n.a.	Increased abundance in COVID-19 patients
Influenza (H7N9 infection)	2021	Murine (C57BL/6) model	Gut/oral gavage (live and pasteurized; ATCC BAA-835)	Increased abundance during infection Treatment reduced weight loss and mortality in mice Reduced pulmonary viral titers
HIV-1 infection	2021	Human fecal samples	Gut/n.a.	Lowered abundance prior to HIV-1 infection compared to negative controls
Soil-transmitted helminth (STH) infection	2021	Human fecal samples	Gut/n.a.	Increased abundance in children with STH infection
*C. rodentium* infection	2021	Both – murine (BALB/c) DSS-induced colitis model	Gut/oral gavage (live; ATCC BAA-835)	Enriched following hyaluronan treatment Induced protection by induction of goblet cells
	2024	Murine (C57BL6/J) Post-infectious irritable bowel syndrome model	Gut/oral gavage (pasteurized; ATCC BAA-835)	Mice were treated 16 days post*-C. rodentium* infection Reduced colonic hypersensitivity and improved intestinal barrier
	2024	Murine (Swiss Webster) Gnotobiotic synthetic microbiota – low and high fiber diet model	Gut/oral gavage (live; ATCC BAA-835)	Increased susceptibility to i *C. rodentium* infection during fiber depletion Reduces pathogen burden in with fiber sufficient diet
*M. tuberculosis* infection	2022	Both – murine(C57BL/6J) antibiotic-treated model	Gut/oral gavage (live; ATCC BAA-835)	Decreased abundance during active *M. tuberculosis* infection Treatment inhibited tuberculosis infection
Trichinellosis (*T. spiralis* infection)	2022	Murine (C57BL/6) model	Gut/oral administration (live and pasteurized; ATCC BAA-835)	Reduced the burden of *T. spiralis*
*C. tropicalis* infection	2022	Murine (C57BL/6J) fecal samples	Gut/ n.a.	Increased abundance in infected (DSS-colitis) mice
Chagas disease (*T. cruzi* infection)	2023	Murine (C57BL/6J and BALB/c) fecal samples	Gut/n.a.	Reduced abundance through disease progression Negative correlation to serum IFN-γ and IL-22
Sepsis	2023	Both – murine (C57BL/6) and piglet model	Gut/oral gavage (live, pasteurized, and purified peptide; strain unspecified)	Reduced abundance in septic patients RKH tripeptide protects against lethal sepsis in mice and piglets RKH reduces inflammation and organ damage in piglets
Severe fever with thrombocytopenia syndrome (SFTS) from *phlebovirus* infection	2023	Both – murine gnotobiotic antibiotic-treated model	Gut/oral gavage (live and pasteurized; ATCC BAA-835)	Increased abundance over course of infection Reduced abundance in deceased patients Protection against SFTS in mice
*L. monocytogenes* infection	2023	Murine (C57BL/6J) low- and high-fat diet model	Gut/intragastric gavage (live; ATCC BAA-835)	Reduced systemic inflammation Protection from oral and systemic infection
*F. nucleatum* infection	2023	hGECs and murine (BALB/c) model of periodontitis	Oral/oral cavity application (live; ATCC BAA-835)	Inhibited inflammatory effect in hGECs Inhibited *F. nucleatum*-induced periodontitis in mice
Enterotoxigenic *E. coli* (ETEC) infection	2024	Piglet model	Gut/oral gavage (live and pasteurized; ATCC BAA-835)	Reduction of diarrhea following ETEC infection Improved small intestine morphology Reduced expression of ETEC virulence factors
Hepatitis B virus (HBV)	2024	Murine fecal samples (post-FMT)	Gut/n.a.	Increased abundance in mice receiving microbiota from HBV-positive humans
*C. albicans* infection	2025	Both – murine (C57BL/6) antibiotic and immunosuppressant model	Gut/oral gavage (live; ATCC BAA-835)	Modified intestinal microbial community Mitigated *C. albicans* translocation infection Increased tight junctions and systemic inflammation
Gynecological diseases (in pregnancy and non-pregnancy)				
Preeclampsia (PE)	2019	Both – murine model (PE by L-NAME)	Gut/oral gavage (pasteurized; ATCC BAA-835)	Low abundance in PE patients Murine treatment reversed PE symptoms and improved placental health
Gestational diabetes mellitus (GDM)	2020	Human fecal samples	Gut/n.a.	Decreased abundance in individuals with GDM
	2025	Murine GDM model (through high-fat diet and streptozotocin)	Gut/oral gavage (pasteurized; ATCC BAA-835)	Improved glucose intolerance and insulin resistance Alleviated placental inflammation
Fetal growth restriction (FGR)	2022	Murine FGR model	Gut/n.a.	Depletion in male FGR offspring
Pre-term birth (PTB)	2022	Human vaginal samples^*b*^	Vagina/n.a.	Present in 27% of individuals with PTB (compared to 24% with term birth) Present with GBS in 28% of individuals with PTB (compared to 10% with term birth)
Bacterial vaginosis (BV)	2022	Human vaginal samples	Vagina/n.a.	Significantly decreased in patients with BV when compared to healthy controls
GBS vaginal colonization	2022	Both – murine model of GBS vaginal colonization	Vagina/intravaginal inoculation (live; ATCC BAA-835)	Enrichment during GBS murine vaginal colonization Observed with pretreatment and co-inoculation Increased GBS persistence in vaginal lumen
	2025	Both – murine (CD-1) model of GBS vaginal colonization	Vagina/daily intravaginal inoculation (live; ATCC BAA-835)	Increased abundance of GBS in pregnant women with *A. muciniphila* Observed with daily treatment (probiotic) Decreased GBS persistence in the vaginal lumen and cervicovaginal tissue
Mitochondrial dysfunction-mediated placental apoptosis	2023	Human trophoblast cell line and murine (C57BL/6J) PE model	Gut/oral administration (pasteurized; ATCC BAA-835)	Suppressed placental mitochondrial dysfunction and apoptosis
Endometrial cancer	2025	Human vaginal samples	Vagina/n.a.	Enriched in the vaginal microbiota of women with endometrial cancer
Endometriosis	2025	Human vaginal samples	Endometrium/n.a.	High abundance in individuals with chronic endometriosis
*In vitro* fertilization embryo transfer (IVF-ET)	2025	Human vaginal samples	Vagina/n.a.	Correlates positively with high estradiol levels in IVF-ET patients
Human papillomavirus (HPV) infection	2025	Human vaginal samples	Vagina/n.a.	Increased abundance in patients with low to high-grade cervical intraepithelial lesions

^*a*^ Gray shading indicates observational/correlative findings with no follow-up.

^*b*^ Analysis of previously published metadata.

^*c*^ "n.a." indicates no treatment studies were performed.

Recent work in the *Akkermansia* field has established notable differences in genomic diversity (45, 46). As such, species-level heterogeneity is a critical consideration when interpreting *Akkermansia* findings. Work utilizing large-scale genomics first showed an open pangenome and multiple *A. muciniphila* subspecies-level phylogroups (AmI, AmII, and AmIII), each with distinct functional repertoires and a global distribution (45). Further studies isolating and characterizing human strains have proposed additional phylogroups (AmIV and AmV), as well as the subdivision of AmI into AmIa and AmIb (46, 47). With the exception of AmIII, each phylogroup has since been named as follows: AmI, *A. muciniphila* (AmIa, *A. muciniphila* subsp. *muciniphila*; AmIb, *A. muciniphila* subsp. *communis*); AmII, *A. massiliensis*; AmIV, *A. biwaensis*; AmV, *A. ignis*; and AmVI, *A. durhamii* (48–50). Recent work has also identified phylogroup-specific phenotypes, including oxygen tolerance, adhesion, iron/sulfur metabolism, aggregation, and vitamin B_12_ biosynthesis, as well as demonstrated that AmII/AmIV can outcompete AmI in antibiotic-treated mice, indicating that colonization dynamics and host interactions are lineage-dependent (46, 47). It is important to note that the average short-read 16S rRNA sequencing does not meet commonly used thresholds for species-level resolution (48). Accordingly, this review refers to a specific *Akkermansia* species only when its identity is experimentally defined within the primary study, such as in treatment studies, metagenomic analyses, or germ-free murine models with defined microbial communities.

## THE IMPACT OF *AKKERMANSIA* DURING INFECTIOUS DISEASES

### Pathogenic gastroenteritis

A large portion of the work examining the role of *A. muciniphila* on infectious diseases has occurred in the last five years. A 2023 study investigated the role of *A. muciniphila* on *Salmonella enterica* serovar (ser.) Typhimurium infection, where the contribution of both live and pasteurized *A. muciniphila* was examined using a Streptomycin-treated murine model (51). Here, mice were pretreated with *A. muciniphila* for two weeks prior to infection with *S*. Typhimurium. Findings demonstrated that both forms of *A. muciniphila* decreased *S*. Typhimurium fecal and systemic burdens and reduced inflammation through infection (51). *A. muciniphila* was found to promote the expression of host genes involved in gut barrier maintenance, as well as the antimicrobial activity of macrophages. An earlier study examined the impact of *A. muciniphila* on *S*. Typhimurium using a gnotobiotic mouse model with a defined simple human intestinal microbiota (SIHUMI) (10). Here, they examined *S*. Typhimurium infection in the absence or presence of *A. muciniphila* treatment, where they found that *A. muciniphila* exacerbated *S*. Typhimurium infection by inducing intestinal inflammation and disturbing mucus homeostasis (10). A higher burden of *S*. Typhimurium was also found in the mesenteric lymph nodes, suggesting increased pathogenesis in the presence of *A. muciniphila*. While one study found that *A. muciniphila* exacerbated infection, the other found it limited infection, highlighting the importance of the context and the different models used. Importantly, the SIHUMI model uses germ-free mice, which elicit immune abnormalities. As such, the synthetic community may not restore immune function sufficiently to mirror responses seen in conventionally colonized, antibiotic-treated mice, where the immune system is not affected to the same extent (52, 53).

Another recent study examined the role of *A. muciniphila* during enterotoxigenic *Escherichia coli* (ETEC) infection (54). Here, administration of *A. muciniphila* resulted in reduced diarrhea in weaned pigs compared to those who were infected with ETEC without *A. muciniphila*, improved overall small intestinal structure, and upregulated genes involved in antioxidant and intestinal barrier (55). *A. muciniphila* also reduced expression of ETEC virulence genes in the ileum and colon, overall suggesting a protective role against ETEC infection (55). The role of *A. muciniphila* on *Listeria monocytogenes* infection was examined using a murine model (56). In this work, a high-fat (HF) diet was utilized to render susceptibility to *L. monocytogenes*, followed by the administration of *A. muciniphila*. Here, *A. muciniphila* increased resistance to both oral and systemic *L. monocytogenes* infection. Specifically, *A. muciniphila* reduced inflammation of the liver and gut, as well as reduced inflammatory cell infiltration in the ileum. Some of these changes were comparable to those in mice fed a low-fat diet, which were initially less susceptible to *L. monocytogenes* infection. This work provides additional evidence to the protective role of *A. muciniphila* in foodborne illness.

*A. muciniphila* has also been studied in the context of another prominent pathogen of the GI tract, *Clostridium difficile*. Gut dysbiosis resulting from antibiotic usage is a primary risk factor for *C. difficile* infection (CDI) (57). In 2016, examination of a human cohort found that *Akkermansia* spp. was increased in abundance in CDI patients (58). This was further explored when a study found that treatment with *A. muciniphila* prevented weight loss, decreased injury in the colon, and improved inflammation and barrier function in CDI (57). Additional work has further explored this impact using a human colorectal adenocarcinoma (Caco-2) cell line, which is commonly used as a model of the intestinal epithelial barrier (59, 60). This work utilized UV-killed *A. muciniphila* rather than the pasteurized form that many studies use, and examination by scanning electron microscopy (SEM) showed that the morphology of UV-killed *A. muciniphila* remained unaltered. Here, Caco-2 cells were exposed to various combinations of neutralized cell-free supernatant (NCFS) obtained by co-incubating *C. difficile* toxigenic supernatant (Tox-S) with various derivatives of *A. muciniphila* for 1 h, including live, UV-killed, cell-free supernatant (CFS), or extracellular vesicles (EV). This study demonstrated a significantly reduced expression of IL-1β following stimulation with *C. difficile* toxigenic supernatant (Tox-S). Notably, TNF-⍺ was significantly reduced following treatment with live bacteria, UV-killed bacteria, and CFS, but not EVs. This work also demonstrated that the expression of the anti-inflammatory cytokine, IL-10, was significantly increased by all four treatment conditions. Overall, the presence of *A. muciniphila* improved infection outcomes. This work expands the role of *A. muciniphila* during CDI beyond animal models by using a human cell line and further supports the anti-inflammatory role of *A. muciniphila* in CDI.

Work in 2021 and 2024 has examined the impact of *A. muciniphila* during *Citrobacter rodentium* infection (61–63). Initial work demonstrated that hyaluronan treatment improved intestinal inflammation and significantly enriched the abundance of *Akkermansia* spp. (61). The direct effect of *A. muciniphila* was also examined by oral administration, and it was found to enhance the production of mucins by goblet cells and antimicrobial peptides by epithelial cells. Similarly, studies examining the impact of *A. muciniphila* in post-infectious IBS using a murine model of *C. rodentium* infection showed that treatment with pasteurized *A. muciniphila* had beneficial effects, including reduced colonic hypersensitivity and increased intestinal barrier function (63). Together, these findings demonstrate that *A. muciniphila* is an important mediator for protection against *C. rodentium*. Alternatively, *A. muciniphila* was previously shown to be enriched in gnotobiotic mice following fiber depletion and to enhance infection susceptibility to enteric pathogens; however, this role was further explored more directly in the context of *C. rodentium* infection (62, 64). Here, a synthetic microbiota (SM) containing *A. muciniphila* alongside other mucolytic bacteria was used (62). By manipulating the composition of this SM, *A. muciniphila* drives increased susceptibility to *C. rodentium* during fiber deprivation; one mechanism behind this is increased mucus permeability. When *A. muciniphila* was the sole mucin degrader in the SM and the diet was fiber-rich, *A. muciniphila* once more demonstrated a protective role, suggesting a fiber-dependent role of *A. muciniphila* in improving *C. rodentium* infection. Together, these distinct studies underscore that factors, such as dietary context, disease state, and the immunological status of the host (germ-free mice vs conventional mice), critically shape the effect of *A. muciniphila*.

*A. muciniphila* was also recently examined for its involvement in trichinellosis, a parasitic infection caused by the helminth *Trichinella spiralis*. This work examined the effect of B-glucans (BGs) in trichinellosis and found that BGs triggered worm expulsion by modifying the gut microbiota (65). Among these changes, the use of BGs was found to significantly increase the abundance of *Akkermansia* spp. during *T. spiralis* infection. Daily oral supplementation with live and pasteurized *A. muciniphila* decreased the levels of *T. spiralis*, with the pasteurized form working more effectively (65). Additional work explored this effect beyond the role of BGs. Here, mice infected with *T. spiralis* showed nine times increased abundance of *Akkermansia* spp. compared to non-infected controls; however, the direct effect of *A. muciniphila* through treatment was not studied (66). *T. spiralis* is a major cause of cardiac fibrosis (CF) from infective myocarditis (67). Here, it was once more observed that *T. spiralis* infection increases abundance of *Akkermansia* spp.; however, this work also showed that oral administration of live or pasteurized *A. muciniphila* improved helminth-induced CF, demonstrating a causative rather than correlative effect. Quite recently, a study examining the impact of *A. muciniphila* on *Candida albicans* GI tract colonization and subsequent translocation infection was just published (68). Here, mice were infected with *C. albicans* using models of GI colonization and translocation infection, with and without A. *muciniphila* treatment by oral gavage (68). This work showed that *A. muciniphila* greatly reduced *C. albicans* translocation and greatly impacted the intestinal microbial community structure and, consequently, metabolite composition. Treatment with *A. muciniphila* was also shown to reduce pro-inflammatory immune cell infiltration and markers, resulting in an overall decrease in systemic inflammation, and to promote expression of tight junction proteins in the colon (68). Importantly, throughout this work, some of the more profound differences were observed between the translocation infection groups with and without *A. muciniphila*, suggesting a more important role in *C. albicans* translocation infection compared to colonization.

Some additional observational sequencing studies have been performed, including a study in 2018 that examined the impact of schistosomiasis on gut microbiota composition (69). Here, mice were infected with *Schistosoma mansoni* followed by sequencing to examine the associated microbiota in the small and large intestine. Significant expansion of *Akkermansia* spp. was identified in both intestinal sites as well as at two different time points in *S. mansoni* infected mice compared to uninfected controls (69). An additional link to parasitic infection was made in 2021 following examination of soil-transmitted helminths (STH) in humans, which in this study were predominantly caused by roundworm *Ascaris lumbricoides* and the whipworm *Trichuris trichiura* (70). Here, the findings showed a significant increase in abundance of *Akkermansia* spp. in children with STH infection. A few studies have also associated several fungal pathogens with *Akkermansia* spp., including *Mucor circinelloides* and *Candida tropicalis* (71, 72). A study looking into gut microbiota changes during mucormycosis (caused by *M. circinelloides*) found a decrease in abundance of *Akkermansia* spp. in the GI tract of exposed mice (71). Work examining changes in the gut microbiota of *C. tropicalis*-infected mice using a DSS-induced colitis model found that *Akkermansia* spp. were more abundant in the gut of infected mice and were the most affected taxa following *C. tropicalis* administration (72).

### Systemic infection

Dysregulation of the host response during infection can result in sepsis, which is a life-threatening organ dysfunction (73). Recently, in 2023, a study examined the role of *A. muciniphila* in sepsis-induced systemic inflammation and lethality using cecal ligation and puncture (CLP) surgery and LPS injection (74). Following the establishment of sepsis, a decrease in the abundance of *A. muciniphila* in the GI tract was observed in the septic mice compared to the controls. Fecal samples from septic patients were also examined and corroborated this observed decrease in *Akkermansia* spp. (74). Using fecal microbiota transfer (FMT) from septic patients and their controls, the sepsis-associated microbiota was further evaluated in CLP mice. Here, mice that received FMT from patients with a higher abundance of *Akkermansia* spp. exhibited higher survival than those who received lower abundance of *Akkermansia* spp. following FMT. Septic mice pretreated with feces containing high abundance of *Akkermansia* spp. demonstrated decreased levels of systemic inflammation and improved organ injury (74). This study also examined the direct effect of both live and pasteurized *A. muciniphila* on CLP mice and found that only live *A. muciniphila* improved survival time of mice and mitigated organ damage caused by sepsis. This contrast between live and pasteurized *A. muciniphila* led to the examination of *A. muciniphila* culture supernatant, which also protected against sepsis (74). Through metabolomics, this work identified a novel secreted tripeptide Arg-Lys-His (RKH), produced by *A. muciniphila*, and evaluated its function in mitigating sepsis. Here, purified RKH reduced sepsis-induced systemic inflammation in a murine model (74). This effect was also examined using a septic piglet model, where inhibition of TLR4 signaling was identified as the anti-inflammatory mechanism by which RKH functions (74).

In work examining the impact of lymphocytic choriomeningitis virus (LCMV), it was found that *Akkermansia* spp. were significantly enriched in infected mice (75). This was observed only in mice that were infected with a fast-spreading and persistent viral isolate clone 13 (Cl13), compared to a slow-spreading acute viral isolate Armstrong53b (ARM). This bloom was found to occur in a CD8 T-cell-dependent manner, specifically by inducing anorexia in mice (75). The impact of *A. muciniphila* was further explored through oral administration and was found to attenuate certain aspects of the CD8 T-cell response. Specifically, administration modified virus-specific CD8 T-cell response and consequently resulted in delayed viral control in the liver (75). Work has also examined changes in the gut microbiota relative to severe fever with thrombocytopenia syndrome (SFTSV), a tick-borne illness caused by a *phlebovirus* (76). Through examination of human fecal samples, it was shown that *Akkermansia* spp. increased in abundance during the course of SFTSV infection and was found to be decreased in samples from deceased patients. Further study found that surviving patients with increased abundance of *Akkermansia* spp. displayed significantly lowered expression of pro-inflammatory cytokines (76). Using a gnotobiotic murine model, SFTSV-infected mice that were mono-colonized with *A. muciniphila* demonstrated lower viral titers, decreased expression of pro-inflammatory cytokines, and reduced tissue inflammatory lesions. This work also demonstrated that administration of live and pasteurized *A. muciniphila* by oral gavage in an antibiotic-treated murine model of SFTSV infection significantly decreased susceptibility to infection and reduced systemic inflammation (76).

Additional studies have reported trends in *Akkermansia* spp. relative abundance during other systemic infections. For example, in 2019, a study examined the impact of particular microbes that had previously been reported to result in susceptibility or resistance to malaria infection via FMT (77). This was performed using a new model for transgestational malaria infection with *Plasmodium chabaudi chabaud*, which showed that *Akkermansia* spp. was negatively correlated with parasitic burden in gravid mice (77). An additional study examined cerebral malaria (CM), a complication of severe malaria (78). This work assessed *Plasmodium berghei* infection in wild-type and macrophage migratory inhibitory factor (MIF) knockout (KO) mice, as MIF has been shown to be a risk factor in patients with CM. Here, it was found that *Akkermansia* spp. were increased in relative abundance in the gut of *P. berghei*-infected wild-type mice compared to uninfected controls (78). Moreover, *P. berghei*-infected MIF KO mice had decreased amounts of *Akkermansia* spp. in their GI tract compared to infected wild-type mice (78). Here, *Akkermansia* spp. was determined to be a biomarker for *P. berghei* infection in wild-type mice. Another parasite, *Trypanosoma cruzi,* has also been associated with changes in the abundance of *A. muciniphila. T. cruzi* is a flagellate protozoan parasite that causes Chagas disease and can circulate in the peripheral blood (79). Examination of the murine gut microbiome during *T. cruzi* infection of two different murine strains revealed distinct changes in *A. muciniphila*. In BALB/c mice, *A. muciniphila* increased over the course of infection compared to uninfected controls (79). Interestingly, the opposite was observed in C57BL/6 mice. Analysis of serum cytokine concentrations in C57BL/6 mice showed a negative correlation between *A. muciniphila* and the cytokines IFN-γ and IL-22 (79). These studies once again highlight how the impact of *A. muciniphila* can vary across different conditions and different murine models.

Other studies have examined the correlation between *Akkermansia* spp. and viral infections that result in systemic infection. For example, in 2021, a study examined the gut microbiota of HIV-1 seroconverters (SC) compared to negative controls (NC), both pre- and post-testing positive (80). Here, it was reported that *Akkermansia* spp. were significantly lower in abundance in SC individuals at initial visit (prior to testing positive for HIV-1 infection) compared to NCs (80). Following HIV‑1 infection, *Akkermansia* spp. decreased in relative abundance, which the authors state is consistent with prior observations, despite not being explicitly reported in the text (81–83). Lastly, recent work examined the microbiota of individuals infected with chronic hepatitis B virus (HBV) compared to non-infected individuals using FMT (84). Upon analyzing the gut microbiota of FMT mice, an increased abundance of *Akkermansia* spp. was observed in HBV-positive mice. *Akkermansia* spp. were identified as the primary contributors to the separation between HBV-positive and HBV-negative mice in terms of beta-diversity (84).

### Pulmonary infection

Recent work has begun to show an association between *Akkermansia* spp. and outcomes in respiratory tract infections. In 2021, a study examined alterations in the gut microbiota of mice infected with avian-origin influenza A (H7N9) (85). While H7N9 is an avian virus, this zoonotic pathogen has been linked to a significant amount of human cases and deaths. Here, *Akkermansia* spp. were identified to be significantly enriched during H7N9 infection, suggesting a link between *Akkermansia* spp. and H7N9 pathogenicity (85). This study further examined this effect through supplementation with live or pasteurized *A. muciniphila* by oral gavage during H7N9 infection. Here, *A. muciniphila* significantly improved survival of infected mice and reduced weight loss. Interestingly, pasteurized *A. muciniphila* provided more protection than live *A. muciniphila*. Pasteurized *A. muciniphila* reduced proliferation of H7N9 virus and vastly improved pathology in the lungs of mice by reducing inflammatory cell infiltration, alveolar atrophy, and fibrosis (85). Lastly, this work demonstrated that *A. muciniphila* improved the innate immune response to H7N9 infection, suggesting an anti-influenza function for *A. muciniphila* that is dependent on its anti-inflammatory and immunoregulatory roles (85).

*Mycobacterium tuberculosis* is an airborne bacterial pathogen that is the leading cause of tuberculosis (TB) in humans (86). In 2022, a study examined the impact of active TB infection on the gut microbiome and found that *Akkermansia* spp. were significantly reduced in infected individuals when compared to healthy controls (86). Here, it was found that *Akkermansia* spp. provided anti-TB protection and reduced tumor necrosis factor (TNF), suggesting that regulation of the TNF response may be a mechanism by which it modulated TB susceptibility. This study further examined the impact of live *A. muciniphila* on TB, finding that mice gavaged with *A. muciniphila* exhibited reduced hemorrhage and pathological impairment in their lungs (86). *A. muciniphila* was also found to induce high levels of palmitoleic acid, a mechanism that contributed to anti-TB protection.

Additional observational studies have correlated *Akkermansia* spp. to outcomes in severe acute respiratory syndrome coronavirus 2 (SARS-CoV-2) infection in humans (87). In 2021, a study examining the gut microbiota of patients infected with SARS-CoV-2 found significant alterations in gut microbiome composition of patients with coronavirus disease 19 (COVID-19) compared to uninfected individuals (87). Here, they found elevated abundance of *Akkermansia* spp. in patients with COVID-19 (1.06%) compared to those without COVID-19 (0.77%) and a positive correlation between *Akkermansia* spp. and plasma levels of IL-6 (87). This relationship was examined more recently in 2025, and the same increase in *Akkermansia* spp. was observed in patients with COVID-19 (88). It is important to note that, while SARS-CoV-2 is a virus that infects the respiratory tract, extrapulmonary symptoms have been observed in patients with COVID-19, including gastrointestinal issues, making the study of *A. muciniphila* during COVID-19 infection a unique and relevant advance (88).

### Periodontitis

Periodontitis is a chronic inflammatory disease that encompasses numerous tissues within the oral cavity, including the gingiva, alveolar bone, cementum, and periodontal ligament. In 2019, a study examined the effect of *A. muciniphila* on inflammation driven by *Porphyromonas gingivalis* using *P. gingivalis*-induced calvarial abscesses and an experimental periodontitis (EIP) model (89). Here, they determined that administration of live *A. muciniphila* by daily oral gavage, alongside *P. gingivalis*, greatly decreased soft tissue inflammation and calvarial bone destruction resulting from *P. gingivalis* infection. In EIP, the administration of *A. muciniphila* resulted in decreased alveolar bone loss (89). *In vitro* studies also showed that bone marrow-derived macrophages that were infected with *P. gingivalis* and *A. muciniphila* showed increased IL-10 and decreased IL-12 production when compared to *P. gingivalis* alone. Similarly, human gingival epithelial cells (hGECs) exhibited increased expression of junction integrity markers when *P. gingivalis* was co-infected with *A. muciniphila* compared to *P. gingivalis* alone. Lastly, co-culture of *A. muciniphila* and *P. gingivalis* resulted in reduced expression of virulence factors, known as gingipains, in *P. gingivalis* and upregulation of Amuc_1100 (encoding a pili-like protein) in *A. muciniphila* (89).

These findings were expanded to explore the impact of purified Amuc_1100 on macrophage polarization during *P. gingivalis* infection, showing that *A. muciniphila* reduced alveolar bone loss and that Amuc_1100 was sufficient to drive this phenotype (90). The administration of live *A. muciniphila* or Amuc_1100 resulted in a significant increase in anti-inflammatory M2 macrophages and a decrease in inflammatory M1 macrophages, suggesting that the decrease in bone loss is associated with a pro-resolutive M2 phenotype switch (90). Lastly, an additional study from 2022 found that pasteurized *A. muciniphila* was also sufficient to reduce *P. gingivalis*-induced periodontal destruction and inflammation (91). Notably, they found that only oral administration of the pasteurized *A. muciniphila* prevented tissue destruction when compared to gastric gavage, suggesting the requirement of local administration for this effect.

Another important causative agent of periodontitis is *Fusobacterium nucleatum*, which is known to copolymerize with other periodontal pathogens (92). This work found that *A. muciniphila* can inhibit the growth of *F. nucleatum* in both its planktonic and biofilm states, as well as suppress the expression of *F. nucleatum* virulence genes. Oral administration of *A. muciniphila* reduced the inflammatory effect of *F. nucleatum* on hGECs and inhibited *F. nucleatum*-induced periodontitis in a murine model, resulting in a significant reduction in bone loss and inflammatory markers in murine periodontal tissue (92). Together, these studies demonstrate a new role for *A. muciniphila* in the prevention and treatment of periodontitis.

## EMERGING ROLES OF *AKKERMANSIA* IN HOST GYNECOLOGICAL HEALTH

The link between the presence and abundance of *Akkermansia* spp. to gynecological and pregnancy outcomes marks a new field of *Akkermansia* research. In 2019, abundance of *Akkermansia* was first linked to the pregnancy-specific multisystem disorder preeclampsia (PE) (93). Through examination of a human cohort, researchers found that patients with PE demonstrated significant depletion of *Akkermansia* spp. in their gut microbial composition. In 2022, this effect was further observed in an additional human cohort and further examined through administration of *A. muciniphila* in a rat model of L-NAME (a nitric oxide synthase inhibitor)-induced PE. This work found that treatment improved placental and fetal outcomes through the regulation of M2 macrophage polarization and spiral artery remodeling in the placental bed (94). It was also found that patients with PE and healthy late pregnancies (LP) could be distinguished by their abundance of *Akkermansia* spp. and the firmicute *Oscillibacter* with 89.7% accuracy, and when paired with blood pressure (BP) and urine protein (UP), this accuracy increased to 98.98%. These findings propose the potential role of *Akkermansia* spp. as a biomarker in PE pregnancies. This was also explored using a murine L-NAME PE model, in which administration of *A. muciniphila* resulted in reduced BP and UP, increased number of pups, and higher pup and placental weights, overall improving PE symptoms in mice (95). This relationship has been further expanded by an independent group examining the effect of EVs, as well as pasteurized *A. muciniphila*, on PE (96, 97). In addition to confirming that live *A. muciniphila* improved PE symptoms, this study demonstrated that oral gavage with EVs also greatly improved outcomes in a murine L-NAME PE model; however, no comparison was made between live *A. muciniphila* and EVs to evaluate efficacy (96). The biodistribution of EVs was examined by fluorescence, demonstrating for the first time the presence of *A. muciniphila* EVs in the placental tissue and fetuses just 5 h post-administration by oral gavage (96). An additional study has shown that pasteurized *A. muciniphila* exhibits a comparable therapeutic effect to live bacteria during L-NAME induced PE in mice by improving the gut barrier, promoting placental angiogenesis, and restoring endothelial function (97). More recently, this group has also demonstrated that pasteurized *A. muciniphil*a inhibits mitochondrial dysfunction-mediated placental apoptosis, an adverse effect of PE (98). Overall, in the context of PE, *A. muciniphila* has been shown to significantly enhance fetal development and growth and ameliorate placental pathology, highlighting for the first time its potential positive role in supporting pregnancy health.

*Akkermansia* spp. were first described in the context of gestational diabetes mellitus (GDM) in 2020, following fecal microbiome analyses of women with and without GDM (99). This work showed that the relative abundance of *Akkermansia* spp. was negatively associated with blood glucose levels. This relationship was further explored by an independent group using murine models of GDM; however, this was in the context of supplementation with xylooligosaccharide (XOS), a prebiotic fiber that has been shown to greatly improve gut health. This initial study found that XOS increased fecal abundance of *Akkermansia* spp. (100). This group further probed the role of *A. muciniphila* by directly treating GDM mice with *A. muciniphila* alone or in combination with XOS by oral gavage. Here, *A. muciniphila* alone improved blood glucose and serum insulin levels, but together with XOS, had a more profound synergistic effect toward improving insulin resistance. Lastly, a group recently examined the effect of pasteurized *A. muciniphila* using a murine GDM model independent of XOS (101). This work found that oral administration of pasteurized *A. muciniphila* greatly enhanced glucose homeostasis and improved placental inflammation. Additionally, the purified protein Amuc_1100 also promoted these anti-inflammatory properties sufficiently (101). Furthermore, recent work found that *Akkermansia* spp. were depleted in adult male mice that underwent fetal growth restriction (FGR) during gestation (102). This depletion was independent of a control or high-fat diet that was fed during the weaning period, suggesting a specific consequence of FGR during fetal development. However, this work was observational, and the direct impact of *Akkermansia* spp. on FGR has not been further explored.

Studies have shown that microbial transit between the GI and female genital tract (FGT) greatly influences human health (103). The rectum has been found to serve as a reservoir for numerous members of the FGT microbiota, and a common hypothesis is that a portion of the vaginal microbiota is seeded by members of the gut microbiota (104). This has been further suggested by evidence that the oral use of probiotics alters the vaginal microbiota in both healthy individuals and individuals with predispositions (105–107). Given the importance of *A. muciniphila* in the GI tract, it can be argued that it is also important to understand the role it may have in the FGT. The first account of *A. muciniphila* in the FGT was in 2016, when examination of endometrial fluid from infertile patients undergoing *in vitro* fertilization (IVF) identified *Akkermansia* spp. in the endometrial microbiota (108). Expanding on this, a study in 2025 examining the endometrial microbiota of participants found increased abundance of *Akkermansia* spp. in participants with chronic endometriosis (1.65%) when compared to those without (1.1%) (109). Importantly, the direct impact of *A. muciniphila* has not been further explored in the context of IVF or endometriosis, as these studies are currently only correlative. The first connection to *Akkermansia* spp. and the vaginal microbiome was made in 2022, during the study of Group B *Streptococcus* (GBS) vaginal colonization of mice (110). GBS colonization led to changes in the murine vaginal microbiota, and the emergence of certain bacterial taxa was predictive of GBS colonization, including *Akkermansia* spp. Interestingly, this work also demonstrated that intravaginal pretreatment with *A. muciniphila*, as well as co-inoculation of GBS and *A. muciniphila*, increased GBS persistence in the murine vaginal tract (110). Recently, additional work expanded on mechanisms by which *A. muciniphila* impacts GBS both *in vitro* and *in vivo*. This work demonstrated that *A. muciniphila* co-aggregated to GBS and increased GBS adherence to human vaginal epithelial cells (111). During a murine model of GBS vaginal colonization, daily intravaginal treatment with *A. muciniphila* reduced GBS burden in the cervicovaginal mucosa (111).

Additionally, the presence of *Akkermansia* spp. in the vaginal microbiota was examined using a previously published cohort of pregnant women (110). In this work, *Akkermansia* spp. were found in 24% of individuals with term birth (TB) and 27% with pre-term birth (PTB) (110). GBS and *Akkermansia* spp. were found to co-occur in 10% of TB individuals and 28% of PTB individuals. These findings suggest that co-detection of GBS and *Akkermansia* spp. was more common in individuals who delivered pre-term compared to those who delivered at term. Importantly, the role of *A. muciniphila* in pre-term birth has not been further explored, and therefore, this relationship is currently observational. *A. muciniphila* was also observed in the vaginal microbiota of a separate pregnancy cohort (111). Here, it was found that *A. muciniphila* was detected in 4.1% of vaginal swabs from pregnant individuals, and of those individuals with *A. muciniphila*, 87.1% were also positive for GBS, suggesting increased likelihood of GBS presence when *A. muciniphila* is present (111). Further, GBS abundance was higher in samples positive for *A. muciniphila*.

Additional work examining the vaginal microbiota of individuals with bacterial vaginosis (BV) found that *Akkermansia* spp. were more abundant in healthy subjects than those with clinical symptoms of BV (112). Higher levels of *Akkermansia* spp. in the vaginal microbiota were found to be positively correlated with increased estradiol in individuals undergoing IVF-embryo transfer, suggesting it may serve as a biomarker for high estradiol levels (113). Of note, no correlation was found between *Akkermansia* spp. and IVF outcomes. Lastly, a study examining the vaginal microbiota of individuals with human papillomavirus (HPV) infection and either no cervical intraepithelial neoplasia (NILM), low (LSIL), or high-grade squamous intraepithelial lesions (HSIL) found that *Akkermansia* spp. were more abundant in individuals with LSIL and HSIL compared to those with NILM (114). Notably, this work raised the question of whether the presence of *A. muciniphila* in these disease states could represent a repair function, considering its beneficial role in many other disease states.

## FUTURE DIRECTIONS FOR *AKKERMANSIA* RESEARCH AND CONCLUDING REMARKS

While murine studies are crucial to providing mechanistic insight, it is important to note that they do not fully recapitulate human physiology, including, but not limited to, the complexity and composition of the human microbiota and differences in human immunity. Therefore, the primary studies reviewed herein that examine treatment with *A. muciniphila* should be interpreted as mechanistic and hypothesis-generating to avoid extrapolation in the absence of supporting human intervention studies. As such, we emphasize the need for controlled human intervention studies to validate mechanisms and associations observed in murine studies. Currently, a search for “*Akkermansia muciniphila*” on the government clinical trials website brings up a total of 50 studies of varying statuses. Of these, 20 interventional studies have been completed that examined diabetes, obesity, and various cancers. Among the other studies, 10 are actively recruiting, 14 are active but not yet recruiting, and five have an unknown status. Some of these current clinical trials are looking at the role of *A. muciniphila* in relation to postmenopausal osteopenia (NCT05348694), GDM (NCT06794723, NCT06548828), acne (NCT06992154), and bipolar disorder (NCT05762887). The range in topics of these trials highlights the demand to expand our understanding of *A. muciniphila* as a therapeutic agent.

As noted throughout this review, extensive studies have assessed the direct role of *A. muciniphila* on disease through supplementation, including in the context of infectious and gynecological disease. This has been done with both live and pasteurized *A. muciniphila*, as well as other bacterial components, including: Pili (Amuc_1100), EVs, aminoacyl tRNA synthases, P9 secreted protein, RKH tripeptide, and ornithine lipids (115–119). Likewise, there are many companies that have gained much popularity in the last few years for producing probiotic supplements containing *A*. *muciniphila*, including Pendulum Therapeutics, GEZORN, Akkermy, and Infiniwell, which use live *A. muciniphila* in their products, and the Akkermansia Company, which uses pasteurized *A. muciniphila*. Importantly, most murine research has examined the use of *A. muciniphila* via oral administration, typically by gavage as a direct line to the gut, or in a few cases, simply localized to the oral cavity during periodontal disease. To date, only one study has suggested a probiotic role through intravaginal administration of live *A. muciniphila* (111). The beneficial effects of pasteurized *A. muciniphila* have been observed across numerous disease contexts, including enteric bacterial infections; helminth and viral infections; and periodontal disease, as well as pregnancy-associated complications, such as PE and GDM, often demonstrating efficacy comparable to that of live bacteria in preclinical models. Collectively, these findings highlight the translational and therapeutic potential of pasteurized *A. muciniphila* in conditions where the administration of live bacteria may be unfavorable.

**Fig 2:**
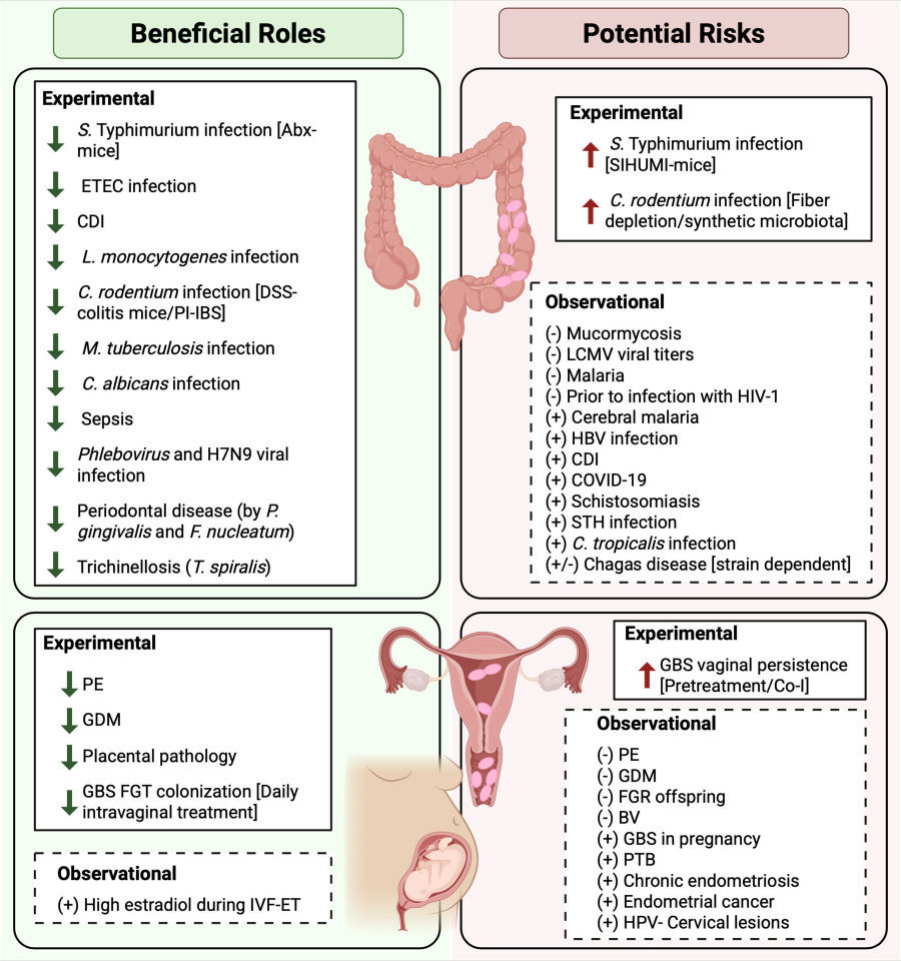
Various roles of *Akkermansia* in infectious diseases and gynecological health. This diagram illustrates the different roles of *Akkermansia* spp. that are discussed in this review. Solid boxes indicate findings from experimental studies that used *A. muciniphila*. Dashed boxes indicate correlative findings from observational studies, where (+) indicates increased abundance or enrichment and (−) indicates decreased abundance or depletion. Abbreviations: HIV-1, human immunodeficiency virus 1; CDI, *Clostridium difficile* infection; COVID-19, coronavirus disease 2019; STH, soil-transmitted helminth; PE, preeclampsia; GDM, gestational diabetes mellitus; FGR, fetal growth restriction; BV, bacterial vaginosis; GBS, Group B *Streptococcus*; PTB, pre-term birth; HPV, human papillomavirus; IVF-ET, *in vitro* fertilization embryo transfer. Created using Biorender.com.

Since its identification in 2004, *A. muciniphila* has garnered significant attention as a keystone commensal with multifaceted roles in host pathophysiology. Within the infectious diseases covered throughout this review, *Akkermansia* spp. demonstrate a consistent propensity to function in a protective manner, demonstrating an array of beneficial outcomes during disease (Fig. 2). When beneficial, a commonality among findings includes improved epithelial barrier integrity through enhanced tight junction protein expression and increased goblet cell abundance and mucin production. Additionally, there is extensive evidence of reduced inflammation that is illustrated through decreased tissue damage in many cases, as well as anti-inflammatory immune modulation. Moreover, there is also enhanced immunity against infection through increased production of antimicrobial peptides following treatment with *A. muciniphila*. In many cases, there is also a direct reduction in pathogenic burden. Yet, it is important to note that its role can sometimes be context-dependent, and *Akkermansia* spp. can either exacerbate or ameliorate infection outcomes depending on factors, such as murine model, microbiota complexity, diet, and even the form of *A. muciniphila* treatment. For example, in the case of *S*. Typhimurium infection, the role of *A. muciniphila* greatly depends on the murine model. On the one hand, *A. muciniphila* treatment in a gnotobiotic model resulted in increased inflammation and *S*. Typhimurium burden. On the other hand, an antibiotic-treated murine model demonstrated an improved gut barrier and decreased pathogenic burden following *A. muciniphila* treatment. These studies emphasize that reported negative effects primarily emerge in constrained settings (germ-free hosts with simplified consortia) and should be interpreted with caution. This complexity is further highlighted through the review of observational/correlative studies that do not test the direct effect of *A. muciniphila* through supplementation or treatment. As such, it is hard to conclude whether depletion or expansion of *Akkermansia* spp. results in disease or is a consequence of disease progression. For example, it is possible that parasitic infection, leading to expansion of *Akkermansia* spp. (such as during schistosomiasis or STH infection), is a compensatory response, potentially resulting from inflammation-associated changes in the GI mucus layer. Yet, it is also possible that increased abundance of *Akkermansia* spp. in the gut leaves the host more susceptible to these infections. In contrast, depletion of *Akkermansia* spp. during systemic disease (such as in the case of sepsis or SFTSV) could suggest that decreased abundance of *Akkermansia* spp. increases susceptibility to infection. Alternatively, the systemic inflammatory state associated with these infections, and its potential impact on the GI environment, may reduce the capacity of *Akkermansia* spp. to colonize or survive.

The limited expanse of published literature identifying *Akkermansia* spp. in the FGT potentially suggests that studies using deeper sequencing or shotgun metagenomics may be better for detecting low abundance taxa of the FGT in the future. While emerging research on *Akkermansia* spp. pertaining to gynecological health, and more specifically its presence in the FGT, continues to develop, there are extensive questions that remain. With the exception of studies on PE and GDM, a large portion of the work reviewed here linking *Akkermansia* spp. to gynecological health is observational. Thus, although it is found to be increased in cases of PTB, chronic endometriosis, endometrial cancer, and cervical lesions, it is unknown whether this is causative or compensatory, as discussed above. Due to its role as a gut commensal, there has been extensive work examining the effect of *A. muciniphila* on the gut microbiota, but knowledge on how it is interacting or modulating the vaginal microbiota is limited. Likewise, the role of mucin degraders in the FGT is considerably less studied when compared to those in the GI tract, which is an important area for future study (120). Mechanistic insights are needed to advance the field of *A. muciniphila* research toward new applications and to further its potential as a therapeutic agent. While its functional versatility offers exciting opportunities for therapeutic development, its context-dependent effects caution against broad application without understanding the molecular mechanisms of interactions between *A. muciniphila* and the host, the microbiota, and pathogenic microbes. Forthcoming work should prioritize understanding these interactions to more safely harness the potential of *A. muciniphila* as a next-generation probiotic across diverse domains of health. As such, rigorously designed, controlled-trial intervention studies are needed to determine whether findings from preclinical murine models translate to clinically meaningful outcomes in humans.
